# Study on the impact of COVID-19 epidemic and agent disease risk simulation model based on individual factors in Xi’an City

**DOI:** 10.3389/fcimb.2025.1547601

**Published:** 2025-05-13

**Authors:** Wen Dong, Henan Yao, Wei-Na Wang

**Affiliations:** ^1^ Faculty of Geography, Yunnan Normal University, Kunming, China; ^2^ Geographic Information System (GIS) Technology Engineering Research Centre for West-China Resources and Environment of Educational Ministry, Yunnan Normal University, Kunming, China; ^3^ Network and Information Center, Yunnan Normal University, Kunming, China

**Keywords:** COVID-19, individual factor, agent model, government macro intervention policy, simulation and prediction

## Abstract

**Introduction:**

Since the first discovery and reporting of the COVID - 19 pandemic towards the end of 2019, the virus has rapidly propagated across the world. This has led to a remarkable spike in the number of infections. Even now, doubt lingers over whether it has completely disappeared. Moreover, the issue of restoring normal life while ensuring safety continues to be a crucial challenge that public health agencies and people globally are eager to tackle.

**Methods:**

To thoroughly understand the epidemic’s outbreak and transmission traits and formulate timely prevention measures to fully safeguard human lives and property, this paper presents an agent - based model incorporating individual - level factors.

**Results:**

The model designates Xi'an—where a characteristic disease outbreak occurred—as the research area. The simulation results demonstrate substantial consistency with official records, effectively validating the model’s applicability, adaptability, and generalizability. This validated capacity enables accurate prediction of epidemic trends and comprehensive assessment of disease risks.

**Discussion:**

From late 2021 to early 2022, it employs a one - to - one population simulation approach and simulates epidemic impacts and disease risks. Initially, using building statistical data in the study area, the model reconstructs the local real - world geographical environment. Leveraging data from the seventh national population census, it also replicates the study area’s population characteristics. Next, the model takes into account population mobility, contact tracing, patient treatment, and the diagnostic burden of COVID - 19 - like influenza symptoms. It integrates epidemic transmission impact parameters into the model framework. Eventually, the model’s results are compared with official data for validation, and it’s applied to hypothetical scenarios. It provides scientific theoretical tools to support the implementation of government - driven prevention and control measures. Additionally, it facilitates the adjustment of individual behavioral guidelines, promoting more effective epidemic management.

## 1 Introduction

The emergence of the severe acute respiratory syndrome coronavirus 2 (SARS-CoV-2) pandemic at the end of 2019 severely disrupted life globally, negatively affecting the mental and physical wellbeing of people around the world (Aldhawyan et al., 2024). The sudden emergence of coronavirus disease 2019 (COVID-19) as a global public health concern not only impacted the global economy but also led to social instability. This pandemic necessitates global unity (Dietz and Brondstater, 2024). Understanding the rapid progression of the COVID-19 epidemic and effectively translating this understanding into government policies has become an urgent requirement for governments worldwide. Rajagopal et al. (2020) developed a SEIRD model with fractional derivatives and validated it using epidemic data from Italy. The results demonstrated that the fractional model exhibited smaller prediction errors compared to the traditional SIR series model. Maier and Brockmann (2020) developed a concise susceptible-infected-recovered-X (SIR-X) model. This model aimed to explore the impacts of isolation measures, containment policies, and unidentified patients, including asymptomatic individuals, on epidemic progression. Truszkowska et al. (2021) constructed an agent-based model. They used it to examine the effects of different vaccination strategies on epidemic prevention and control in New Rochelle, a town in the United States. Beira and Sebastiao (2021) devised a comprehensive protected-susceptible-exposed-infectious-recovered-deceased (s) [PSEIRD(S)] model. They fitted the model using data on the number of infected individuals, deaths, and hospitalizations during the post-Christmas 2020 outbreak in Portugal. The fitting results showed that some model parameters underwent discrete temporal changes, reflecting the multi-phase nature of the epidemic. A comprehensive protected-susceptible-exposed-infectious-recovered-deceased (s) [PSEIRD(S)] model was devised. They fitted the model using data on the number of infected individuals, deaths, and hospitalizations during the post-Christmas 2020 outbreak in Portugal. The fitting results showed that some model parameters underwent discrete temporal changes, reflecting the multi-phase nature of the epidemic.

Building on the basic SIR model of COVID-19 transmission, Dehning et al. (2020) proposed a Bayesian framework based on Markov chain Monte Carlo (MCMC) sampling. This framework was used to characterize key epidemiological parameters, time-varying transmission rates, and potential change points. It also helped identify the optimal timing for intervention effectiveness. Bertrand and Pirch (2021) developed the susceptible-exposed-infectious-quarantined-recovered-deceased (SEIRQD) model. They explored the optimal control of the second-phase COVID-19 lockdown in Morocco and analyzed the impact of optimal control strategies on the pandemic in the country. Wijesekara and Wang (2022) employed the proposed susceptible, transmitted, quarantined, non-diagnosed infected, hospitalized diagnosed infected, recovered, dead, susceptible (SEQIJRDS) model. They predicted mortality rates under various lockdown procedures, vaccination scenarios, quarantine measures, and mask-usage cases. Additionally, they projected hospital resource utilization to identify the most effective interventions that would prevent over-straining hospital resources. Building on the SEIR model of disease transmission, they (Liu et al., 2021) put forward an adaptive model named SEAIRD, which incorporates internal sources and isolation interventions. This model simulates the evolving behavior of SARS-CoV-2 in the United States. Neural networks are applied to enhance the fit of the SEAIRD model. Schlosser et al. (2020) demonstrated that lockdown measures in Germany not only significantly reduced population mobility but also led to a substantial decrease in the long-term connectivity of the mobility network. The study revealed that these structural changes could flatten the epidemic curve and slow down disease transmission. Cacciapaglia et al. (2021) proposed a continuous waveform graph based on the epidemic severity normalizing group framework (eRG). They concluded that understanding the relaxation periods between different epidemic phases is crucial for controlling future major outbreaks. Aleta et al. (2020) constructed an agent-based COVID-19 epidemic dynamics model. By integrating anonymous mobile phone location data from the entire Boston area with census statistics, they found that agents adhered strictly to social distancing requirements for a specific period. Contact tracing, nucleic acid testing, and isolation measures effectively curbed the spread of COVID-19. These measures also alleviated the burden on the healthcare system, enabling it to handle current medical demands.

## 2 Materials and methods

### 2.1 Data collection

This study amassed diverse fundamental epidemiological parameters, including those of the COVID-19 virus. A Python web crawler was utilized to gather and analyze the most recent daily COVID-19 epidemic data publicized by the Xi’an Health Commission on its official website. These data encompasses the number of new cases, cumulative confirmed cases, and new deaths on each given day. The number of deaths are accumulated, and the missing data are further supplemented through the network platform. Through the API interface of AMAP (2022), the geographic coordinates, building categories, and population capacity of various buildings in Xi ‘an, such as houses, schools, hospitals, nursing homes, workplaces, leisure, and entertainment venues, were collected and counted, and the above data were checked and corrected by manual collection on Google Map (Google, 2022). The population data were sourced from China’s seventh national population census in 2020, along with relevant government-released macro-policy data. These were organized into datasets for use in the model. Transmission parameters of the COVID-19 virus, clinical parameters, and patient hospitalization parameters were drawn from research conducted by global scholars and official announcements of Xi’an. Parameters were adjusted in accordance with the actual situation in Xi’an.

### 2.2 Agent model

In this study, the classical SEIR epidemiological infection model underwent improvement. The model’s states were both increased in number and refined. Concurrently, it was integrated with the discrete-event simulation model and extended on the GIS platform, enabling the construction of a comprehensive agent-based model.

Within this study, agents are categorized into Sicken Individual Agent (SIA), Healthy Individual Agent (HIA), and Intervention Agent (VA). The attributes and behaviors of these agents are precisely defined in **Table 1**.

**Table 1:** Agent attributes and behavior definition.

	Agent sort	Common attribute	Concrete attribute	Behavior	Infectious disease influencing factors
Individual agent	SIA HIA	Time attribute	Age	Flow in the location where the model is generated	Location migration
			Sex		
			Whether to become a close contact		
			Whether to become a close contact		
			Whether to become a close contact		Behavior trajectory
			No exposure history (no contact with confirmed patients, suspected cases)		
			Have a history of travel (i.e., have you ever traveled or lived in a place where a confirmed case has been reported)		Social travel
			Have fever, cough, and other symptoms		
	VA	Spatial attribute	When the model is started, an appropriate time attribute value is selected for initialization according to the start and end time of the local government intervention	The VA treats patients with fever and cough	Traffic control
				VA restricts the public activities of agents with a history of exposure and residence	Stay-at-home order
				VA observed and detected agents with exposure history and travel history	No workplace business
				VA controls the traffic of agents with a history of exposure and residence	No gathering, no public activities
				VA conducted standardized isolation therapy for SIA	

During each simulation step (Δs), agents switch locations among the six types of places generated by the model, guided by their own requirements. The model assigns distinct propagation parameters to different locations. For simulated travelers, the flow of various vehicles between different regions is factored in. Travel modes are interconnected through the Haversine equation—an algorithm calculating distances between two points using latitude and longitude data. This mimics real-world scenarios where people select travel modes based on distance and time constraints.

Six typical locations are denoted as Q={1,2,3,…., q}, while residents in the study area are C={1,2,3,….}, A transfer function fk: C → Q is defined using the semi-positive vector formula. Here, k∈{F, B, S, Rt, Hp} is used to allocate generated inhabitants to various locations. The function fk sends each agent i ∈C to its corresponding location q. Locations q include homes (F), public places (B), schools (S), nursing homes (Rt), and hospitals (Hp). Given that an agent may not be connected to all locations, when agent i has no connection with location q, we denote it as fk(i) = ∮, and Cq represents the total number of agents connected to location q.

The Haversine formula (Baskar and Anthony Xavior, 2021) is as follows:


d=2Rarcsin (√sin2 ()+cos(lat2) cos(lat1) sin2 ()) (1)

This paper builds an Agent model across three dimensions: individuals, settings, and the epidemic itself. At the individual level, Agents represent real-world people as embodied entities. The setting dimension pertains to the environment where Agents interact and carry out actions. Regarding the epidemic, it encompasses the age distribution of hospitalized patients, the proportion of ICU hospitalizations, and the age-specific mortality ratio. This paper conducts research at three levels. At the city level, Xi’an is chosen as a representative city for epidemic outbreaks. At the community level, research focuses on family units. Using census data from the study area, data such as the total number of household accounts, age ratio, total population, age distribution of household heads, proportion of single-parent families, proportion of childless families, and proportion of families with the elderly are integrated with official data for model calculation, demonstrating local community structure characteristics. Building data in the study area is retrieved via the AMAP API interface to recreate the real-world environment. Starting from community characteristics and combining the total number of working people, working hours, and the proportion of different transportation methods, the local population mobility model is analyzed and simulated.

**Figure 1:**
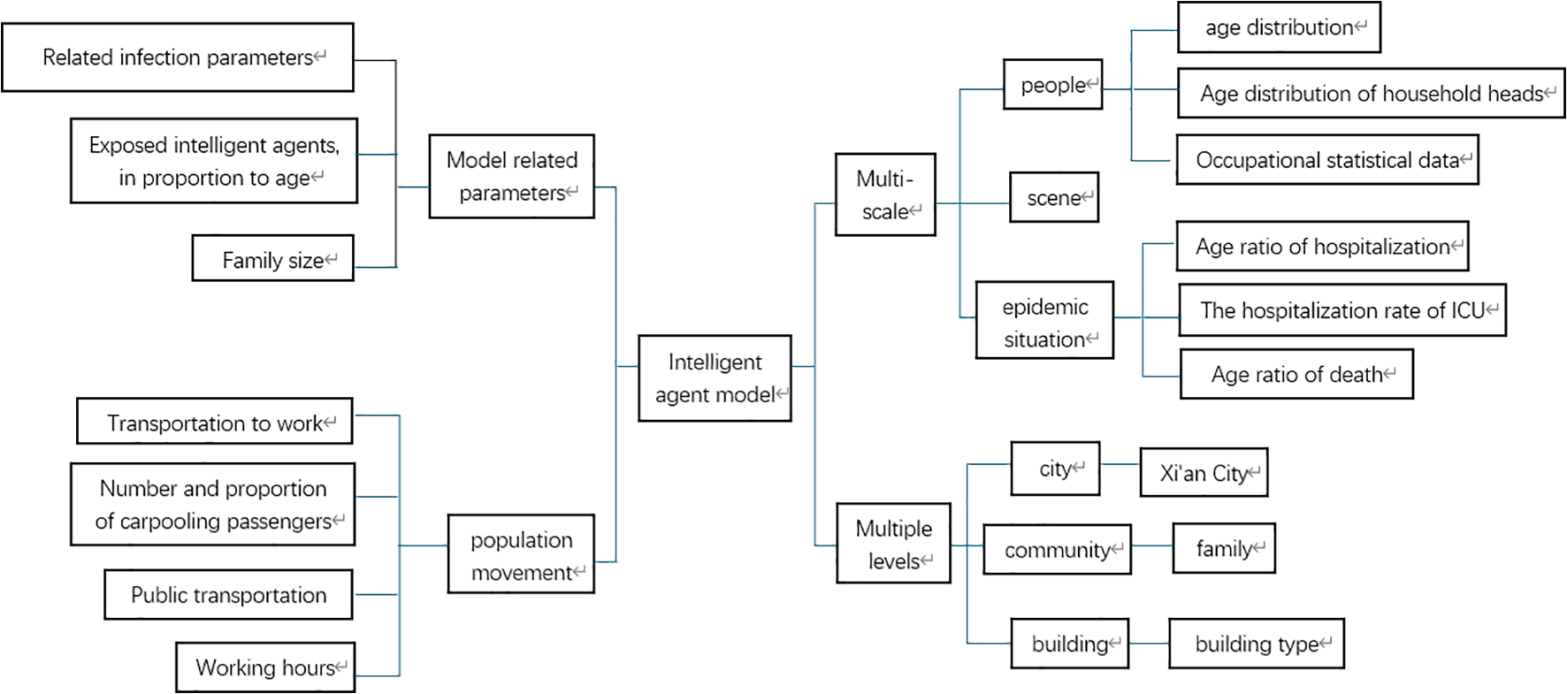
Model architecture diagram.

Relevant model parameters are integrated. Infection-related parameters, family size, and age-related proportions of agents after exposure are incorporated into the SEIR model as code. This enables their specific impact on the spread of infectious diseases. The model architecture is illustrated in **Figure 1**.

## 3 Result

### 3.1 Simulation and verification of agent model based on Xi’an City

The model amasses and organizes data including geographical latitude and longitude coordinates, building types, population capacities, and similar information for various buildings such as houses, schools, workplaces, hospitals, and leisure and entertainment venues in all districts of Xi’an, China. The population data come from Xi’an’s 2020 seventh national census. With this data, a population-related model is established. The number of students and school employees is retrieved from statistical results publicized by the Xi’an Education Bureau. The number of hospital employees and hospitalized patients is sourced from statistical announcements by the Xi’an Health Bureau.

**Figure 2:**
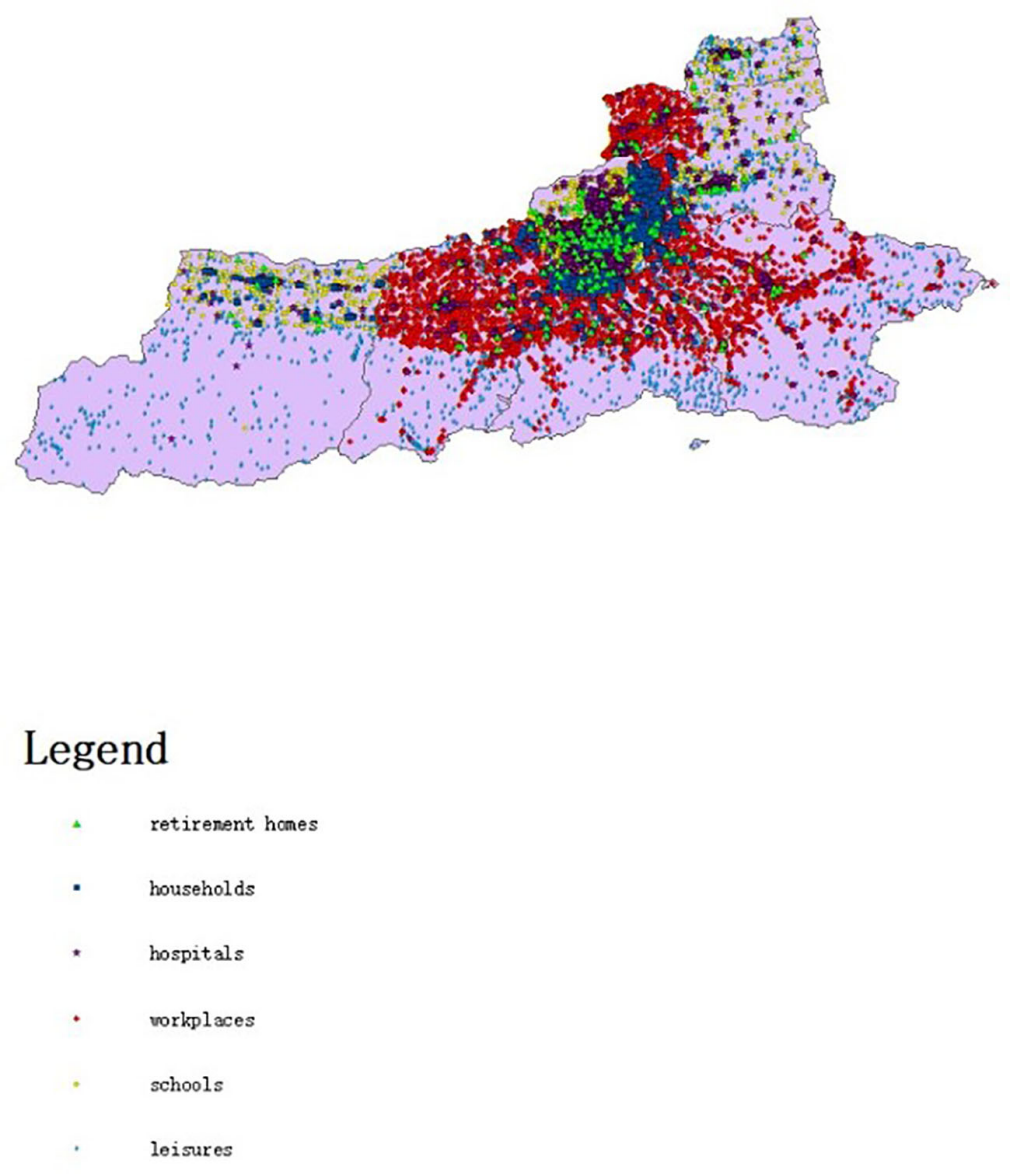
Map of different types of buildings in Xi’an.

**Figure 3:**
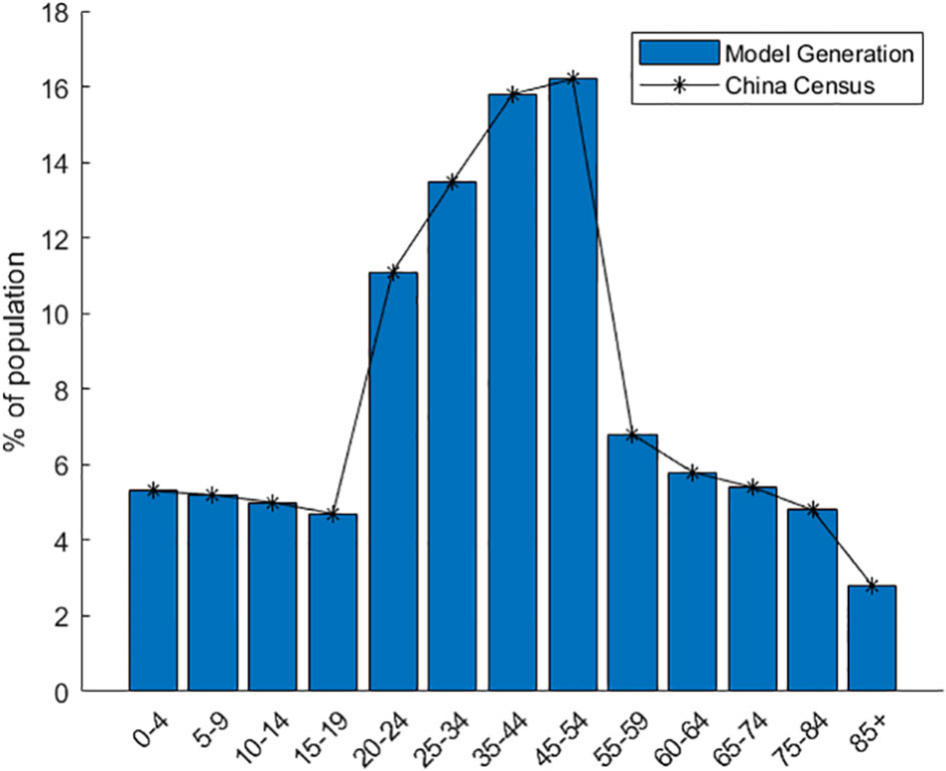
Distribution of population age characteristics created by the model.

The model assigns housing to individual agents statistically. It bases this on data from the seventh national census, like the number of existing families, average family size, and housing vacancy rate in Xi’an. The model generates 13,078,200 agents and forms 5,577,852 households. At the start of model operation, the entire generated population of Xi’an is initialized as susceptible individuals. A specific number of agents are set in the exposed state. During subsequent simulations, agents change states. When the model is initialized, **Figure 2** presents the relevant locations of the created buildings. **Figure 3** shows the age distribution generated by the model using census data.

The study applies evenly distributed random sampling to perform nucleic acid testing on agents within the susceptible population. Agents can be in diverse states, such as the healthy state, undergoing testing, or receiving treatment. Both individuals with influenza-like symptoms similar to confirmed infection and exposed persons must undergo nucleic acid testing. The daily volume of nucleic acid testing is scheduled according to actual timelines. Once the test results are available, decisions on hospitalizing patients are made based on positive or negative outcomes, although there are also some false-negative and false-positive cases.

Specifically, nucleic acid detection is conducted on individuals who were within the same 800 m × 800 m spatio-temporal range as confirmed patients. The study does not involve explicit and specific tracking. Case detection is achieved on an average-significance basis, and nucleic acid detection for a particular agent is determined through even distributed random sampling.

According to test results, infected individuals are classified into two categories: asymptomatic carriers and confirmed cases. The model arranges routine hospitalization for ordinary confirmed cases. For agents with deteriorating conditions, the model assigns ICU treatment. For agents in centralized isolation, when they exhibit symptoms of COVID-19 infection, their status changes to routine hospitalization or ICU treatment. For agents requiring treatment, their treatment status can be adjusted based on clinical observations. Considering the patient’s age and the probabilities of routine and ICU hospitalization, initial treatment plans are derived from clinical data (Eubank et al., 2020; Verity et al., 2020). For patients admitted to the ICU, their recovery status is recalculated based on ICU mortality (Verity et al., 2020). After diagnosis, the treatment type of agents will be changed. Since cured and deceased patients no longer influence the spread of COVID-19, the model will remove them. The model also takes into account a category of agents with a high mortality rate: patients who have not undergone nucleic acid testing yet still require ICU treatment. In line with our policy, the number of such agents is set to zero in the model.

When susceptible agents contract the common cold or seasonal influenza, they are quarantined due to symptoms resembling those of COVID-19 (Verity et al., 2020). If the nucleic acid test result is negative, the quarantine is lifted (Verity et al., 2020).

The model simulated the spread of COVID-19 in Xi’an from 1 December 2021 to 29 January 2022—from the onset of the epidemic to the reopening of Xi’an. This aimed to demonstrate the model’s practicality. In this study, three types of COVID-19-related data released by the Xi’an Municipal Health Commission were selected: the daily number of newly confirmed cases, the cumulative number of confirmed cases, and the total number of deaths. Using these data, weekly new cases and deaths in the study area were extracted to validate the model.

For instance, by altering the daily nucleic acid detection rate and the number of initially infected people in the model, it was observed that the infection rate in public places decreased during Xi’an’s complete lockdown. Conversely, the infection rate increased after Xi’an reopened. In line with the actual situation, the number of agents undergoing nucleic acid testing daily changed over time.

**Figure 4:**
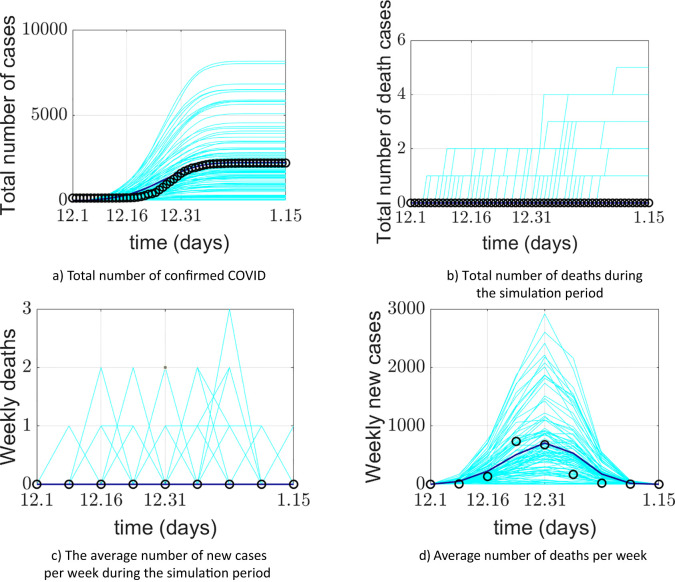
Comparison between the COVID-19 epidemic in Xi’an simulated by the model and the official reported data. **(a)** Comparison of cumulative confirmed cases; **(b)** comparison of cumulative deaths; **(c)** comparison of the average weekly number of new cases; **(d)** comparison of average weekly deaths. The cyan lines in the figure represent 100 implementations of the model, the blue lines represent the average of the model implementations, and the o represents the official reported data.

The model simulation was run 100 times. **Figure 4** presents the model validation results. The model’s output data were verified and compared with Xi’an’s real-world data from four aspects: a) the total number of COVID-19 confirmed cases during the simulation period, b) the total number of deaths during the simulation period, c) the average weekly number of new cases during the simulation period, and d) the average weekly number of deaths during the simulation period. The total number of COVID-19 confirmed cases represents the sum of false positives detected during model operation and all positive agents. Some diseased agents died because they could not be admitted to the ICU due to medical resource constraints. Introducing weekly averages helps avoid spurious fluctuations caused by uneven data collection.

The model’s output regarding the total number of COVID-19 confirmed cases, weekly new cases, and weekly deaths aligns closely with the actual data reported by the Xi’an Health Commission. This validates the model’s effectiveness in predicting daily new cases. The simulated 95% confidence interval for the cumulative number of confirmed cases was (1526.059982, 1076.640018), while the actual 95% confidence interval was (1458.295568, 990.1377655). The 95% confidence interval for the simulated weekly new confirmed cases was (470.6553997, 117.5946003), compared to the actual 95% confidence interval of (464.8929787, 117.3570213). The simulation and prediction accuracy for the cumulative number of confirmed cases and mask-wearing rate reached 100%.

The model exhibits generality. To apply it to different cities, parameters need to be determined based on each city’s specific circumstances, enabling city-specific simulation and prediction. During a certain period, the cumulative number of confirmed cases output by the model is slightly higher than the actual official data. This could be attributed to the frequency of official data reporting. It may also be due to the implementation of patient nucleic acid testing and active contact tracing at the start of model operation, corresponding to the early stage of the epidemic. Thus, under the model-provided practical scenario of patient nucleic acid testing, discrepancies between simulated and actual data may occur. Regarding weekly new cases and simulations under a 100% mask-wearing rate scenario, spikes in real-world data may be associated with variations in hospital reporting frequency or delays.

### 3.2 Simulation and effect evaluation of agent epidemic prevention scenario

The virus exists in droplets or aerosols. When air carries and spreads particles contaminated with the virus, people can inhale them directly, resulting in infection. Infected individuals exhale virus-laden particles while breathing, communicating, coughing, sneezing, or singing. This easily infects those in their vicinity. Therefore, wearing masks stands as a crucial measure to prevent virus spread.

**Figure 5:**
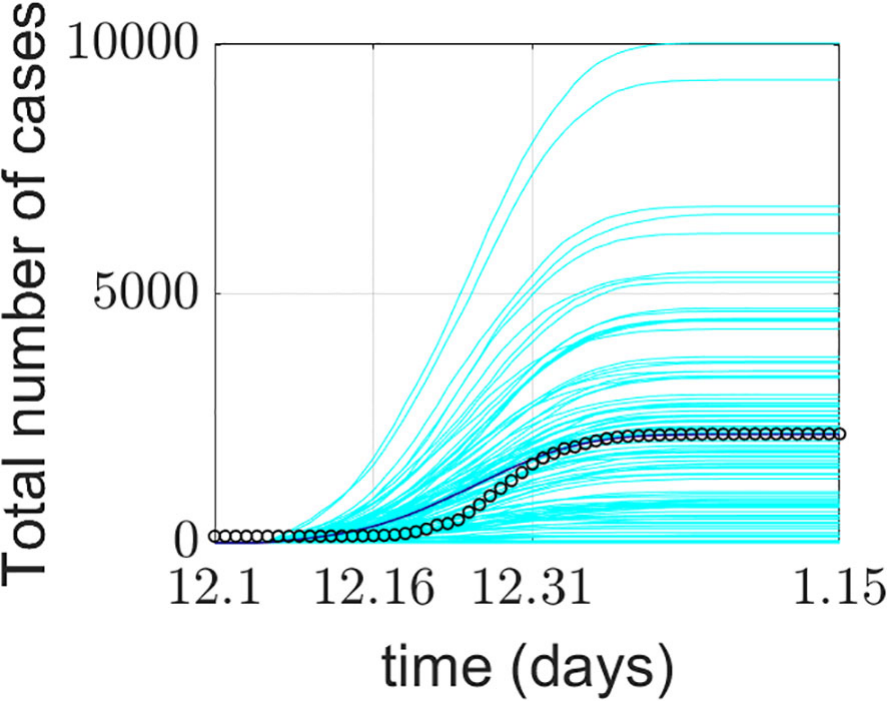
Cumulative number of confirmed cases in Xi’an with masks.

**Figure 6:**
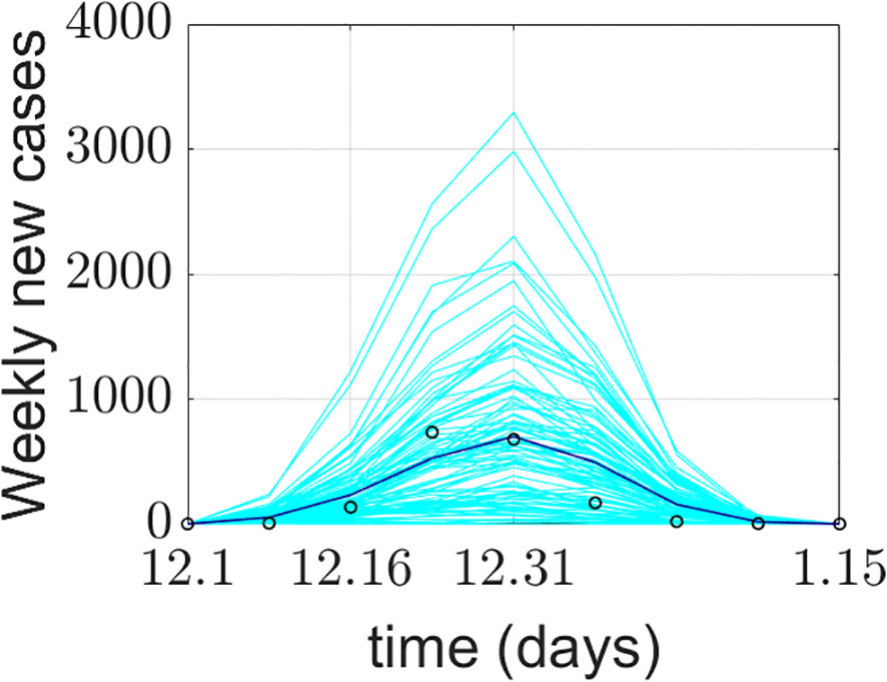
Number of new cases per week in Xi’an with masks.

To demonstrate the model’s value and the impact of mask wearing on the COVID-19 epidemic, the model simulated a scenario where all Xi’an residents wore masks in public places during the outbreak. This means that the mask-wearing rate was set at 100%, and a 1-m social distance was maintained. According to the health-behavior survey of Xi’an citizens, 90% of them wear masks when going out. **Figures 5** and **6**, respectively, display the simulated COVID-19 situation in Xi’an regarding the cumulative number of confirmed cases and the weekly new cases, compared with official data under a 100% mask-wearing rate.

**Figures 5** and **6** depict the simulated COVID-19 situation and official reported figures in Xi’an during scenario simulations involving mask-wearing and social distancing: a) comparison of cumulative confirmed cases and b) comparison of cumulative deaths. In the figures, cyan lines denote 100 model simulations. The blue line represents the average of model implementations, while circles (o) represent officially reported data.

When the mask-wearing rate hits 100%, both the cumulative number of confirmed cases and the weekly increment in the model’s output decline slightly although not substantially. This may stem from some individuals’ casual approach to the epidemic, leading to imperfect execution of mask wearing.

## 4 Discussion

In late December 2019, Wuhan, a city in Hubei Province, emerged as the first location where the COVID-19 virus was detected and reported. Shortly thereafter, the virus began to spread swiftly, reaching numerous countries worldwide (Marcel et al., 2020). This caused significant harm globally. To effectively contain the COVID-19 outbreak in China, it is essential to carry out efficient testing, promptly trace patient contacts, and identify related cases. Targeted prevention and control and rational public health intervention strategies will play a positive role in reducing the spread of COVID-19 (Aleta et al., 2020; Marcel et al., 2020; Unwin et al., 2020; Adriana et al., 2021). Identifying infected individuals and tracing their subsequent contacts represents a crucial aspect of the testing process. To address these challenges, it is necessary to gain a more in-depth understanding of community composition, the location and context of COVID-19 outbreaks, and people’s daily habits (Singh et al., 2020; Heidarzadeh et al., 2021). To maximize the alignment between the experimental environment and the population characteristics and geographical environment of the study area, this research drew on relevant data from the seventh national population census of Xi’an. Data including the total number of permanent residents in Xi’an, the total number of household accounts, the age ratio, the age-distribution characteristics of household heads, the average family size, and the proportions of childless families, single-parent families, and families with the elderly were collected.

The population agents of Xi’an in the model were generated using data that reflect the local community structure. Based on these data, family units were created for the agents. Subsequently, family members were assigned to each family, with the granularity reaching individual level. This approach aimed to achieve one-to-one modeling of residents in the study area and reconstruct the community characteristics of the study area to the greatest extent possible. Accurate and comprehensive map data are essential for reconstructing the real-world geographical environment of the study area. On the one hand, the map data were sourced from the basic map data of the study area. On the other hand, leveraging AMAP and Baidu Map is crucial for acquiring detailed statistical data on all buildings in the study area. These data include building types, names, precise locations, and their latitudes and longitudes. The data are generated onto the study area’s basic map using Point of Interest (POI) data, facilitating the creation of a comprehensive study area map. This, in turn, maximally restores the real-world geographical environment of the study area.

To conduct simulation research on the impact of macro-intervention behavior on epidemic transmission and disease risk, considering individual factors, it is essential to gather data on individual influencing factors and relevant government macro-policy data. Individual-factor data encompasses an individual’s age, occupation, travel mode, history of traveling in areas with suspected cases, contact with confirmed patients, presence of symptoms like fever, cough, and fatigue, population density at their location, and implementation of traffic control and public-activity restrictions. Government macro-policy data cover containment and quarantine measures, promotion of mask-wearing and avoidance of social activities, and closure and reopening of schools and workplaces. Beginning with the unique community characteristics of the study area and integrating the total number of working individuals and the data on the proportion of different transportation modes that they select for commuting, the local population’s movement patterns and behavior trends can be analyzed. When residents commute to work, attend school, or visit the hospital, their movements contribute to crowd mobility, thereby forming the crowd movement model. Given that infection rates vary across different settings, this paper primarily focuses on residents at home, during crowd movement, and in locations such as schools, hospitals, and workplaces. By integrating the infection rates specific to these relevant settings, the simulation of the epidemic transmission process is completed. For confirmed patients, the model classifies them into general hospitalization and ICU treatment categories based on predefined age parameters. Confirmed patients undergoing treatment in general hospital wards will experience improvement and recovery, influenced by factors such as the COVID-19 recovery time. For patients receiving ICU treatment, the model predicts the number of deaths among confirmed patients by considering relevant parameters like the ICU treatment mortality rate and patient age. The model no longer includes recovered and deceased confirmed patients in its calculations. Subsequently, a comprehensive agent model is constructed. In this study, a model is developed within a simulated spatial environment that closely resembles the real-world geographical landscape. This achieves a seamless integration of agent entities and the geospatial environment.

Building on existing research, Zhou et al. (2022)and his team effectively demonstrated the transmission process of COVID-19. They integrated data from mobile network operators with the space–time simulation technology of gravity models. Through this approach, they identified four primary transmission modes. These modes are constrained by the specific spatial layout and geographical location of the city. In this study, the simulation of human behavior is not only accurate at the individual level but also provides highly accurate spatiotemporal representation for each individual (Ferguson et al., 2005, 2006; Perez and Dragicevic, 2009; Ajelli et al., 2010; Perra and Gonçalves, 2015; Aleta et al., 2020), In the agent-based model, various physical locations are considered, including enterprises, hospitals, residences, nursing homes, and schools. Simultaneously, by integrating the unique attributes of the community, this research delves into the behavior patterns of local residents and population movement trends. This approach aims to overcome the limitations imposed by relying on specific spatial layouts and geographical locations. By integrating population density and spatial density across different regions to predict outbreak times, the corresponding mathematical model is developed to achieve epidemic early warning. An agent-simulation prediction model is designed and implemented, considering the unique spatial structures and community characteristics of Chinese cities and the interactions of government macro-intervention measures. In the control and prevention of disease spread, the construction and analysis of mathematical models are of crucial importance. The compartment model serves as the foundation for understanding the complex dynamics of epidemics (Jiao et al., 2020). This model can be employed to analyze the impact of influencing factors, transmission routes, and population susceptibility on disease progression. It offers scientific theoretical support for the rational formulation of prevention strategies. Koo et al. (2020) utilized a model simulating the influenza epidemic to explore the impact of four interventions on the transmission of the COVID-19 epidemic in Singapore under three scenarios with different basic reproductive numbers. Compared to previous studies, this paper’s advantage lies in its straightforward coding approach. This enables more efficient presentation of the effects of various macro-interventions. It facilitates public health institutions in quickly and promptly evaluating current strategies and provides direction for subsequent policy adjustments. The compartment model is also an invaluable tool for understanding epidemics and evaluating pre-conceived strategies (Baldea, 2020; Della Rossa et al., 2020; Estrada, 2020; Gilbert et al., 2020; Vespignani et al., 2020). This research model builds upon the SEIR model. By incorporating the patient’s death state, nucleic acid detection state, and treatment state, and integrating with the discrete-event simulation model, it expands based on GIS. This results in the formation of the entire agent model. Currently, many agent models focus on specific settings such as university campuses. For instance, Gressman and Peck (2020) investigated an agent model centered on a university campus. This model was used to explore small-scale micro-environments, like the strategies for universities to reopen during the epidemic, or to construct agents for simulating an entire country as the research context. This paper conducts numerical simulations in a medium-scale environment to explore interactions between agents themselves and between agents and their environment. Tatapudi et al. (2021) developed an agent model based on population and social data from an urban community in the United States with 2.8 million residents. They used it to study the impact of vaccine prioritization strategies on curbing COVID-19. A large number of experimental studies have validated the advantages of agent models. These studies have demonstrated their technical feasibility and expandability in cross-scenario research (Adler et al., 2020; Gopalan and Tyagi, 2020; Keskinocak et al., 2020; Kuzdeuov et al., 2020; Kerr et al., 2021). By integrating relevant elements, this paper addresses the scarcity of medium-scale agent simulations in existing research. It also develops an agent model suitable for Chinese cities, incorporating the unique characteristics of Chinese community structures. This model can more accurately and comprehensively reflect the distinct lifestyles of Chinese residents. Moreover, it enables refined population representation when simulating large-scale activities, making it highly relevant to the epidemic outbreak, prevention, and control processes in China. Although this paper has certain limitations, its fitting results are generally consistent with official data. This provides scientific reference for public health departments to adopt more comprehensive, rigorous, and detailed measures to safeguard public health.

## 5 Conclusions

Acknowledging the complexity and uncertainties within human society, human behavior, and the continuous mutation of the COVID-19 virus, this paper gathered 60 days’ worth of epidemic case data from Xi’an. The data collection period extended from late November 2020 to early 2021 and encompassed cumulative confirmed cases, cumulative deaths, daily new cases, and daily deaths. Taking Xi’an’s epidemic situation as a case study, the research incorporated geographic information technology. It delved into the attributes and characteristics of typical clustered epidemic scenarios. This investigation relied on population census statistical data, which enabled one-to-one population modeling. It also made use of basic geographic data including longitude and latitude information of homes, schools, hospitals, public areas, nursing homes, Xi’an’s basic map data, and epidemic big data. Spatial structures closely related to the COVID-19 transmission path, such as homes, schools, hospitals, nursing homes, public areas, and leisure and entertainment venues, were identified. One-to-one high-precision modeling of geographical buildings was carried out. This created a physical environment for simulating the “human” (agent) and “epidemic” (virus transmission process) scenarios of COVID-19. During the modeling process, the model offers two distinct nucleic acid detection methods based on residents’ travel status: hospital-based detection and home-isolation detection. The model precisely tracks and performs nucleic acid testing on individuals who have had spatio-temporal intersections with patients. Patients are classified into general hospitalization, ICU hospitalization, and home isolation (after treatment completion) based on the severity of their symptoms and hospitalization requirements. Special scenarios, such as agents working in schools, hospitals, and nursing homes, are modeled separately. The daily number of nucleic acid tests varies over time. Moreover, accounting for the additional burden of nucleic acid testing imposed by influenza patients with virus-like symptoms, Xi’an was simulated to undergo complete lockdown, isolation, and reopening. This aimed to simulate the impact of different government policies and measures on virus transmission during the movement and interaction of urban residents. In addition, a prediction model for the spread of COVID-19 in urban space was proposed. This model incorporates individual factors, geospatial structures, and macro-intervention behaviors. The dynamic transmission patterns of the disease were explored within spatial distribution units, focusing on the interaction between agents and intervention agents. Finally, the model’s results were compared with official epidemic data released by the Xi’an Health Commission.

This study effectively developed a COVID-19 agent-based disease risk prediction model. The model can capture the influence of individual factors on the epidemic and the impact of macro-intervention measures. It utilizes multi-source data, such as influenza statistics, building data, and population data. By comprehensively applying the agent-based model, it enables long-term and dynamic simulation and prediction of the COVID-19 epidemic transmission trend in Xi’an, the disease risk faced by residents, and the effectiveness of intervention strategies. This model serves as a reference for the government to anticipate epidemic development, optimize the allocation of prevention and control resources, and evaluate prevention and control measures.

## Data Availability

Publicly available datasets were analyzed in this study. This data can be found here: https://www.kingcounty.gov/depts/health/covid-19/data.aspx.
